# Psychological and behavioural impact of returning personal results from whole-genome sequencing: the HealthSeq project

**DOI:** 10.1038/ejhg.2016.178

**Published:** 2017-01-04

**Authors:** Saskia C Sanderson, Michael D Linderman, Sabrina A Suckiel, Randi Zinberg, Melissa Wasserstein, Andrew Kasarskis, George A Diaz, Eric E Schadt

**Affiliations:** 1 Department of Genetics and Genomic Sciences, Icahn School of Medicine at Mount Sinai, New York, NY, USA; 2 Health Behaviour Research Centre, Department of Epidemiology and Public Health, University College London, London, UK; 3 Department of Clinical Genetics, Great Ormond Street Hospital, London, UK; 4 Icahn Institute of Genomics and Multiscale Biology, Icahn School of Medicine at Mount Sinai, New York, NY, USA; 5 The Children's Hospital at Montefiore and the Albert Einstein College of Medicine, Bronx, NY, USA

## Abstract

Providing ostensibly healthy individuals with personal results from whole-genome sequencing could lead to improved health and well-being via enhanced disease risk prediction, prevention, and diagnosis, but also poses practical and ethical challenges. Understanding how individuals react psychologically and behaviourally will be key in assessing the potential utility of personal whole-genome sequencing. We conducted an exploratory longitudinal cohort study in which quantitative surveys and in-depth qualitative interviews were conducted before and after personal results were returned to individuals who underwent whole-genome sequencing. The participants were offered a range of interpreted results, including Alzheimer's disease, type 2 diabetes, pharmacogenomics, rare disease-associated variants, and ancestry. They were also offered their raw data. Of the 35 participants at baseline, 29 (82.9%) completed the 6-month follow-up. In the quantitative surveys, test-related distress was low, although it was higher at 1-week than 6-month follow-up (Z=2.68, *P*=0.007). In the 6-month qualitative interviews, most participants felt happy or relieved about their results. A few were concerned, particularly about rare disease-associated variants and Alzheimer's disease results. Two of the 29 participants had sought clinical follow-up as a direct or indirect consequence of rare disease-associated variants results. Several had mentioned their results to their doctors. Some participants felt having their raw data might be medically useful to them in the future. The majority reported positive reactions to having their genomes sequenced, but there were notable exceptions to this. The impact and value of returning personal results from whole-genome sequencing when implemented on a larger scale remains to be seen.

## Introduction

Whole-genome and exome sequencing are increasingly used in clinical, research, and commercial contexts. Whole-genome sequencing (sequencing of most of the euchromatin DNA in a genome) and exome sequencing (sequencing of the protein-coding parts of the genome) are used for diagnosing rare diseases,^1, 2^ large-scale research into rare and common diseases,^3^ and are available commercially direct to consumers.^4^ These technologies are more comprehensive and detect more types of DNA variation than targeted genetic tests and single-nucleotide polymorphism (SNP) arrays. However, the huge volume of data produced by whole-genome sequencing in particular raises many practical and ethical challenges. These include issues around informed consent, data analysis, interpretation of rare variants, and return of results and raw data.^5, 6, 7^

The increasing availability of whole-genome sequencing inside and outside the clinic has prompted debates about its value for ostensibly healthy individuals.^8, 9, 10^ The value of DNA-based information can be thought of as the ratio of benefits to harms, and has traditionally been conceptualized as ‘clinical utility' (usefulness in the clinic and impact on clinical end points).^11^ However, genomic information may also have personal and social value, for example, as entertainment, learning, or a way to relate to others.^12, 13^ Understanding how individuals react to personal results will be key in assessing how they themselves perceive the potential utility or value of whole-genome sequencing.

Although studies have begun exploring issues related to the value of personal genomic sequencing for healthy individuals,^14, 15, 16, 17^ these largely focus on health-related results. SNP-based studies have similarly focused primarily on health-related results,^18, 19, 20^ often for a single disease or trait.^21, 22, 23^ Current evidence from SNP-based tests suggests that DNA-based disease risk information has little emotional impact on people,^18^ and does not motivate them to improve health behaviours.^24, 25^ However, SNP-based tests generally do not detect rare variants that may have more important roles in health.

Given that whole-genome sequencing will become the standard in detecting genomic variation in the future, the full scope of potential reactions to the wide range of personal results that may arise from whole-genome sequencing, including rare disease-associated variants, should be explored. Protection motivation theory (PMT)^26^ may provide a useful theoretical framework for the health-related component of this exploration. PMT is a social cognition model that arose out of fear-arousing communications research, and is widely used as a framework for understanding health behaviours. According to PMT, individuals are motivated to engage in health-protective behaviours (‘protection motivation') when they perceive a threat to their health (‘threat appraisal') and when they feel able to reduce that threat through their actions (‘coping appraisal'). SNP-based tests may not elicit strong behavioural responses because individuals appraise the health threat conveyed by the disease risk information to be low, and so are not motivated to act on it. However, rare disease-associated variants may elicit stronger emotional reactions and thus stronger protection motivation and subsequent behavioural reactions.

We therefore explored how healthy individuals reacted to receiving a range of personal whole-genome sequencing results in the HealthSeq project, using the PMT to guide our conceptual framework relating to the health-related results. HealthSeq is an exploratory longitudinal study designed to explore motivations for whole-genome sequencing, satisfaction, and the impact of personal whole-genome sequencing results. In light of the public fascination with aspects of genetics such as ancestry,^27^ non-health as well as health-related results were offered. Raw sequence data was also offered, given current discussions around patients' access to their personal clinical/research data.^28, 29, 30^ As previously reported, most (33/35) HealthSeq participants wanted all the available results, and expressed a range of motivations, including learning personal health-related information, obtaining ancestry information, contributing to research, and satisfying curiosity.^31^ We have also previously reported on participants' satisfaction with the first genetic counselling session.^32^

The overarching aim of this study was to explore how people react to receiving personal results from whole-genome sequencing. The Specific Aims were to explore: (i) the emotional impact and perceived value of personal results from whole-genome sequencing; (ii) the behavioural impact; (iii) whether and how people's reactions vary by results categories (eg, ancestry *vs* type 2 diabetes risk); and (iv) whether and how people's reactions vary according to the personal results they receive within those results categories (eg, increased *vs* decreased risk of type 2 diabetes).

## Methods

This was a mixed-methods longitudinal cohort study in which participants underwent whole-genome sequencing, received personal results, and completed interviews at five time points (T1–T5). Recruitment and T1 procedures have been previously described.^31, 32^ After results were returned at T2 (see the section ‘Return-of-results appointments and reports' below), telephone follow-up interviews were conducted 1 week (T3), 6 months (T4), and 1 year (T5) later. We used PMT to guide our conceptual framework (Supplementary Figure 1) and inform our selection of constructs and measures relating to the health-related results (Supplementary Table 1). For example, we assessed fear (operationalized quantitatively as ‘test-related distress'), and intended and actual behavioural reactions, in response to personal results from whole-genome sequencing. We are not aware of any existing models that provide a framework for both health-related and non-health-related personal results simultaneously, so our formal framework focuses on responses to health-related results only. However, the qualitative topic guide and analysis plan were designed to also explore responses to the non-health-related results, as well as other potentially important psychological constructs and behaviours (see the section ‘Six-month follow-up interviews' below). This paper describes the return-of-results appointment, and presents findings from the T4 interviews and repeated measures from T1 to T4.

### Return-of-results appointments and reports

At the in-person return-of-results appointment (T2), the study genetic counsellor and medical geneticist verified whether the participant still wanted to receive the results categories they had consented to at T1. The personal results were then returned. The participants were given a paper report comprising the following categories of information and interpreted results: sequencing quality; ancestry; physical traits (lactose intolerance, bitter taster type, cilantro taster type, earwax dry/wet type); pharmacogenomics (simvastatin, clopidogrel, warfarin); common polygenic disease risk (type 2 diabetes, coronary artery disease, age-related macular degeneration (AMD)); Alzheimer's disease risk; monogenic disease carrier variants (‘rare carrier variants'); and monogenic disease variants (‘rare disease-associated variants'). The genetic counsellor and medical geneticist systematically reviewed the report with the participant, reviewing each section, providing background and then discussing the meaning of the results. Questions were answered and concerns were addressed for each section before moving to the next. The participants were given the option to receive their raw sequencing data on a portable hard drive. Results sessions lasted approximately 1 h depending on the quantity of results and participant concerns. Immediately after the counselling session, the participants completed the T2 interview with the research coordinator. On completion of the study (ie, after all follow-up interviews had been completed), the results reports were reviewed and all rare variants that had been classified as pathogenic or likely pathogenic were re-interpreted, to examine whether any classifications had changed in the intervening time.

### Six-month follow-up interviews

The T4 telephone interview schedule comprised two parts. Part one was a topic guide containing seven broad questions plus suggested prompts. The questions were developed by a multidisciplinary team and informed by PMT and other relevant literature. The questions were designed to explore reactions to personal results from whole-genome sequencing; understanding and recall of results; and satisfaction with procedures and materials. This paper focuses on reactions to personal results from whole-genome sequencing. The relevant questions in the topic guide included: How are you feeling about your decision to get your genome sequenced? How did your genome sequencing results make you feel? Did your results influence your behaviour in any way? If yes, how? Have you shared or intend to share your results with anyone? If yes, with whom and why? Part two of the interview schedule contained valid, reliable measures of depression,^33^ anxiety,^34^ quality of life (QoL),^35^ positive experiences;^36^ PMT-related measures were fear (test-related distress^36^) and lifestyle behaviours.^37^ Interviews were audio-recorded and transcribed verbatim for qualitative analysis. Responses to closed-ended questions were entered into a database for quantitative analysis.

### Analyses

A thematic framework approach was developed by SCS and SAS for the analysis of the transcripts from part one of the interviews.^38, 39^ The initial broad themes were guided by the literature and the overarching objectives, and included two sets of themes: emotional reactions, perceived value, and behavioural reactions; and reactions to results categories and offer of raw data. For the latter set, sub-themes were determined by the categories in the results reports, for example, reactions to ancestry results, reactions to pharmacogenomics results, and so on. For the former set, more differentiated sub-themes were generated in a ‘bottom-up' manner based on an in-depth analysis of 10 interview transcripts: SCS and SAS each analysed five transcripts, individually developed a set of themes and sub-themes based on these analyses, compared the two sets, resolved any differences through discussion, and jointly produced a single codebook. For behavioural reactions, each sub-theme (eg, ‘Communication with family') was further sub-categorized into ‘Action' (has already done this) ‘Intention' (plans to do this), and ‘None' (has not done this and does not plan to). The codebook is available from the authors on request.

SCS used this codebook to code all T4 transcripts using software package NVivo 10. Although not the primary focus here, SCS also read all T2 and T3 transcripts to establish that no key themes were being missed in the T4 transcripts. SAS coded a subset of T4 transcripts and assisted in resolving any uncertainties in coding. A non-exclusive coding approach was used, for example, a text segment could be coded as both ‘negative emotional reaction' and ‘reactions to Alzheimer's disease results'.

To address Specific Aims 1 and 2, the coded text passages were explored and categorized by SCS and SAS into a final characterization of participants' emotional and behavioural reactions overall. To facilitate the analyses for Specific Aims 3 and 4, a tabular thematic framework in Microsoft Excel was used. In this framework, each participant interview corresponded to one row. The columns corresponded to results categories. Each results category (eg, Alzheimer's disease) was represented by two columns. The first column contained the participant's actual result (eg, *APOE* e3/e4). The second column contained their reaction to the result (eg, if a text segment from that participant's interview had been coded as ‘Reactions to Alzheimer's disease result', then that coded text would be entered here). This thematic framework approach allowed us to explore whether different themes emerged for different categories of genomic results, as well as for different personal results within those categories. It also allowed us to read across the rows, facilitating understanding of individual participants' stories.

For the measures in part two of the interview, variables were described using means, standard deviations, and frequencies. Changes over time were assessed using repeated-measures analysis of variance (ANOVA) and the Wilcoxon signed-rank test. All statistical analyses were conducted using software package SPSS v.20 (IBM, Chicago, IL, USA).

## Results

### Socio-demographic characteristics

Twenty-nine (82.9%) of the 35 participants completed T4 and were included in the analyses. As Table 1 shows, the participants were 41% female, aged 26–68 years, 79% White non-Hispanic, and 52% had annual household incomes over $150000.

### Results returned to participants

Seven participants received results reports that included one or more pathogenic (P) or likely pathogenic (LP) rare disease-associated variants results. Participant 15 was informed he had an LP variant associated with Brugada syndrome and was one of two participants who received *APOE* e4/e4 results indicating increased Alzheimer's disease risk. Two participants received genetic risk scores (GRSs) indicating a likelihood ratio of AMD greater than 2.0. Most (25/29) were informed they had at least one variant classified as P/LP for carrier status. Table 2 summarizes each participant's results, and shows which P/LP disease-associated variants results were re-classified, for example, as variants of uncertain significance (VUS), after the study had ended.

### Emotional and behavioural reactions overall: themes and example quotes

#### Positive emotional reactions

Most participants expressed positive feelings about having had their genomes sequenced. Six major themes emerged: (i) feeling happy or relieved; (ii) it was interesting; (iii) feeling glad to have contributed to research; (iv) the results or data might be useful to them in the future; (v) the results made them feel more connected to the world; (vi) it was fun. Among the participants who felt happy, some felt this way because they were excited by the science, for example, ‘I'm geeked. I'm still geeked. I was geeked at the beginning. I'm geeked now. I love it. I think it's one of the best things I ever did.' [#32, male, 35–39 years]. See Supplementary Table 2 for sub-themes and additional example quotes.

#### Negative emotional reactions

Although most participants reacted positively, a few expressed negative reactions. Six major themes emerged: (i) concern; (ii) disappointment; (iii) indifference; (iv) confusion; (v) desire for more results; (vi) not relevant to them. Among those who felt concerned, participants talked about the implications for their health, insurance, and their results going into their medical records if they sought medical follow-up. Among those who felt disappointed, for some this was because they had hoped to get information about a specific disease or trait. For others, the results did not meet their expectations and their disappointment was with the general scope of the results, for example, ‘I left the whole process feeling disappointed a little bit in terms of what I actually learned…' [#13, male, 50–54 years]. See Supplementary Table 3 for sub-themes and example quotes.

#### Behavioural reactions

Many participants had acted on their results. Four major themes emerged: (1) shared results with family and friends; (2) sought further information from family or online; (3) shared results with a healthcare provider; (4) made lifestyle changes. Among those who had shared their results with a healthcare provider, the majority had just mentioned them at a pre-scheduled appointment. One had their results put in their medical record. One had made an appointment and had a consultation, including tests and procedures, with a healthcare provider as a direct consequence, and one as an indirect consequence, of their results (see the ‘Rare disease-associated variants' section below). Many participants talked about why they had not shared their results with a healthcare provider. Only a few had made lifestyle changes because of their results. See Supplementary Table 4 for sub-themes and example quotes.

### Reactions to specific results: themes and example quotes

#### Rare disease-associated variants

Two of the seven participants who received P/LP rare disease-associated variants results were concerned about and acted on their results. In the first case, participant 15 (who received the LP Brugada-associated variant result) said, ‘It was pretty concerning to me because it is a sudden cardiac death mutation, so it's one of those things that, it really can strike at any time, and it's, so I was scared for that reason.' [#15, male, 25–29 years]. The result directly prompted him to make an appointment and have a consultation, including tests and procedures, with a healthcare provider: ‘So I was able to meet with [the cardiologist], and he did the EKG, which didn't find anything pathological… After talking to him, I was reassured…'. He anticipated that the result might be empowering in the future: ‘It was stressful in the short-term… I think going forward I'll be concerned about the risk of sudden cardiac death, but I think hopefully this information will be empowering.' Over time, he had become less concerned with the Brugada-associated variant, and more concerned about his Alzheimer's disease e4/e4 result, saying, ‘I think I heard the APOE4 APOE4 high risk of Alzheimer's, and kind of didn't think much about that at that point in time because my concern was more on the immediate risk of death, but… I've probably thought more and more about the Alzheimer's than about Brugada.' [#15, male, 25–29 years]. In the second case, participant 7 was concerned enough about her two rare cardiac disease-associated variant results to seek further information from relatives about family cardiac health history, which then prompted her to make an appointment and have a consultation with a healthcare provider. See Table 3 for further details.

#### Alzheimer's disease

Both the participants who received *APOE* e4/e4 results were concerned. One of the seven participants who received e3/e4 results was confused and unclear about the health implications. See Table 3 for further details.

#### Multifactorial diseases

The multifactorial (polygenic) type 2 diabetes and coronary artery disease results generally had little impact on participants. Of the two participants who had received GRSs above 2.0 for AMD, one had shared their result with their healthcare provider at a pre-scheduled appointment, and the other intended to make an appointment with an ophthalmologist but had not done so yet.

#### Rare carrier variants

The rare carrier variant results generally had little impact, although a few participants said they might be useful to them or their children for reproductive decision-making in the future. Some felt these results were not relevant to them, and that they should have opted out of this section.

#### Pharmacogenomics

The participants found the pharmacogenomics results interesting. Some felt these results might be useful to them clinically in the future. Some had a desire for more results in this category, and would have liked to know about more medications in general, and about antidepressants specifically.

#### Physical traits

Most participants did not react to the physical traits results, although a few said they were fun.

#### Ancestry

Many participants found their ancestry results very interesting, and some found them fun. Many said that they had shared these results in particular with family and friends, for example, ‘I was telling my parents about it, some of my friends. Mostly people were interested about the ancestry part.' [#26, male, 35–39 years]. Some said their ancestry results made them feel more connected to the world, for example, ‘…It was surprising, and fascinating, and just enriching of my sense of myself and my place in the world.' [#18, male, 60–64 years].

#### Raw sequence data

Several participants felt that, even though most could not open their raw sequence data files, their raw data might be useful to them clinically in the future, for example, ‘Or if God forbid I get cancer, myself, then I would probably want to revisit that data.' [#07, female, 55–59 years].

### Quantitative outcomes

**Figure 1:**
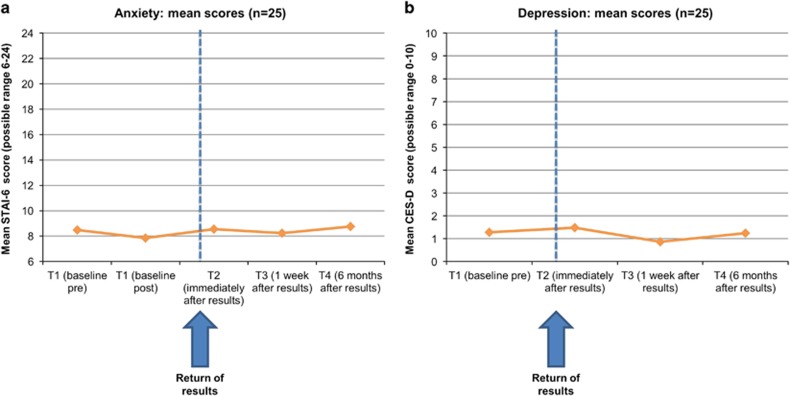
Anxiety and depression: quantitative outcomes. (**a**) Anxiety; (**b**) Depression.

**Figure 2:**
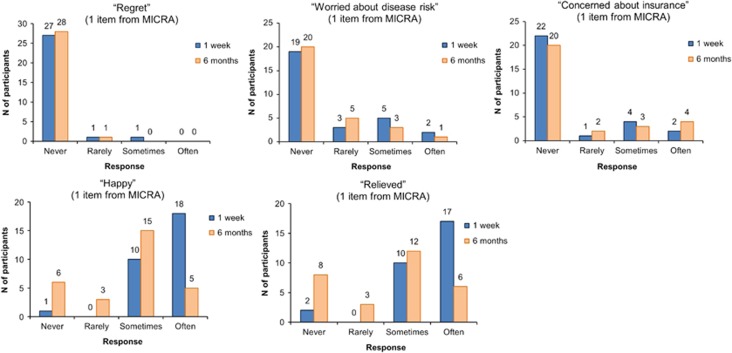
Reactions to personal genomic sequencing results: quantitative outcomes.

**Figure 3:**
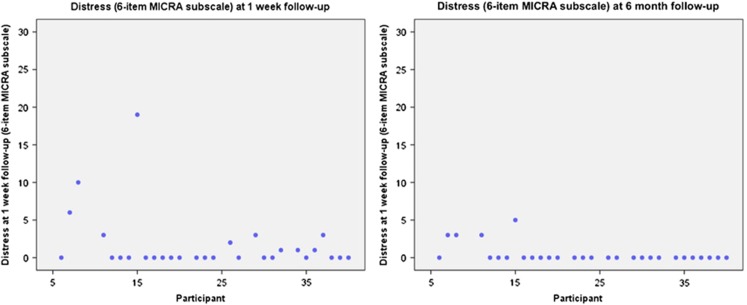
Test-related distress: quantitative outcomes.

At T1, anxiety and depression were low, and most participants said they were in excellent or good health on the QoL measure: no changes over time were detected in anxiety (F=0.19, *P*=0.67), depression (F=0.75, *P*=0.52), or QoL (Z=1.90, *P*=0.058; see Figure 1 and Supplementary Table 5). MICRA positive experiences scale scores were lower at 1-week than 6-month follow-up (lower scores indicated more positive experiences): mean (SD) scores were 2.55 (3.16) *vs* 7.45 (6.83), respectively (*Z*=3.28, *P*=0.001). On individual MICRA items at T3, most said they were sometimes or often happy (28/29) and relieved (27/29) about their results; again, these numbers were slightly lower at T4 (Figure 2). This likely reflects that the MICRA asks participants how often they have felt this way in the past week. MICRA test-related distress scale scores were low: where 0=low distress and 30=high distress, mean (SD) scores were 1.69 (4.00) at T3 and 0.48 (1.27) at T4. Although low at both time points, the scores were lower at T4 than T3 (*Z*=2.68, *P*=0.007). The examination of the scatter plots suggested most participants had low distress but that participant 15 had high distress at T3, which subsided by T4 (Figure 3). As shown in Figure 2, most participants reported they never regretted having their genomes sequenced (27/29 at T3; 28/29 at T4), and never or rarely worried because of their results (22/29 at T3; 25/29 at T4), on individual MICRA items. No changes in lifestyle behaviours were detected (Supplementary Table 5).

## Discussion

In this study, most participants had positive emotional reactions to receiving personal results from whole-genome sequencing. Our findings support the suggestion that personal whole-genome sequencing may have value for individuals in ways that go beyond narrow definitions of health-related clinical utility, and that are consistent with broader notions of personal utility or value.^11, 12, 13^

As observed in previous research using direct-to-consumer and other SNP-based genetic results,^18, 25^ few participants changed lifestyle behaviours as a consequence of their whole-genome sequencing results. This is consistent with PMT, and supported by our qualitative findings: the small increases in diabetes and heart disease risk conveyed in our study were insufficient to elicit health-related fear or protection motivation among participants. This may also reflect well-known difficulties in facilitating lifestyle changes: even complex interventions explicitly designed to change lifestyles achieve modest at best improvements in lifestyles and related outcomes,^40^ and HealthSeq did not provide behaviour change interventions.

Although there was little impact of the complex disease risk results on the participants' emotions or lifestyles, several were worried about and/or acted on other results they received, in particular the rare disease variants results. Taken together, these observations are consistent with PMT: individuals who do not feel threatened by their personal results from whole-genome sequencing (or do not feel they can do anything to reduce their risk) may be unlikely to engage in lifestyle change or to pursue clinical follow-up, but individuals who do feel threatened by their results and who believe they might be able to reduce the health threat may engage in subsequent health-protective behaviours.

It is notable that one participant's focus shifted over several months from his Brugada-associated rare variant result to his Alzheimer's disease *APOE* e4/e4 result, as he processed and dealt with each in turn. This highlights how reactions and counselling needs may change over time, and that counselling protocols may need to reflect this. Ensuring sufficient support is available to people after receiving results is clearly important, but it is not clear yet what the appropriate level of support is nor how to ensure people get the support they need.

A further notable and challenging issue arising in the two cases where ‘likely pathogenic' variants directly or indirectly prompted clinical follow-up, is that these variants were later re-classified as VUSs after study completion. As the knowledge base about rare variants grows, variants are likely to be re-classified over time.^41, 42, 43^ Studies addressing whether and how often people's sequence data can or should be re-interpreted, and how new interpretations should be returned, are needed.

Several participants were unsure about the significance of their results, and uncertain about whether and what follow-up to pursue. This was despite the results being returned in person by a genetic counsellor and medical geneticist, and despite these participants being early adopters with higher levels of education than the general population. The finding that even under these circumstances some participants struggled to understand their results and their implications underscore that significant efforts will be needed to aid comprehension of results in the future as whole-genome sequencing expands to the wider population.

Several participants had mentioned their results to their regular healthcare providers. This is consistent with previous research reporting that over a third of consumers shared their direct-to-consumer genetic results with a physician.^44^ These findings further support the need for an educated healthcare workforce that is prepared to interact with patients about their personal genomic information whether or not that information has been obtained within the clinic.^45, 46^

Recent debates have explored whether research participants have the right to access their own raw data, and whether greater efforts should be made to facilitate this.^28, 30, 47^ Most HealthSeq participants opted to receive and responded positively to having their raw data. Participants' reasons for wanting their raw data were primarily that they felt having it might be of value to them and their physicians in the future. This novel finding provides early evidence that research participants may value having their raw genomic data because they anticipate it may help in their future clinical care.

Strengths of this study include the qualitative methods and the greater age range compared with previous work.^48^ Limitations include that participants were self-selected and had high levels of education and income, so the findings are not generalizable to the wider population. This was a small study, although the sample size was not unusual for qualitative research.^49, 50^ The PMT did not provide a framework for exploring non-health-related results, positive emotional reactions or communication behaviours. A more complete theoretical framework within which to examine the psychological and behavioural effects of results arising from whole-genome sequencing is needed. Development of such a framework may be a particularly valuable focus for future applied psychological research in the genomic sequencing field.

Research participants are key stakeholders in debates about the future directions and value of offering personal results and data from whole-genome sequencing. The findings from this in-depth study with ostensibly healthy individuals suggest the possibility that currently neither the benefits nor harms of personal genome sequencing are significant for most individuals, but that there may be important exceptions to this that warrant further investigation. The impact of returning personal results from whole-genome sequencing results when implemented on a larger scale remains to be seen.

## Figures and Tables

**Table 1:** Socio-demographic characteristics of participants

*Characteristic*	*Participants who completed 6-month follow-up (*N=*29)*
*Gender*	
Male	17 (58.6%)
Female	12 (41.4%)
	
Age (years), mean (SD), range	48.6 (12.1), 26–68
*Age groups*	
18–29 years	3 (10.3%)
30–39 years	5 (17.2%)
40–49 years	5 (17.2%)
50–59 years	9 (31.0%)
60+ years	7 (24.1%)
	
*Race/Ethnicity*	
African American	1 (3.4%)
Asian	1 (3.4%)
Hispanic/Latino	2 (6.9%)
More than one race	2 (6.9%)
White non-Hispanic	23 (79.3%)
	
*Education level*	
Less than Bachelor's degree	0 (0.0%)
Bachelor's degree	10 (34.5%)
Master's degree	12 (41.4%)
PhD/MD/JD	7 (24.1%)
	
*Employment status*	
Employed full-time	24 (82.8%)
Employed part-time	1 (86.2%)
Missing data	4 (13.8%)
	
*Annual household income*	
Below $20000	2 (6.9%)
$20 000–$39 000	0 (0.0%)
$40 000–$59 000	4 (13.8%)
$60 000–$79 000	1 (3.4%)
$80 000–$150 000	6 (20.7%)
Over $150 000	15 (51.7%)
Chose not to answer	1 (3.4%)
	
*Number of individuals in household (besides oneself)*	
Mean (SD), range	1.3 (1.5), 0–5
*Number of biological children*	
Mean (SD), range	0.79 (0.98), 0–3
No children	16 (55.2%)
1 child	4 (13.8%)
2 children	8 (27.6%)
3 children	1 (3.4%)

Note that all the data are expressed as number (%), unless otherwise indicated.

**Table 2:** Participant characteristics and personal results from WGS

					*Additional details regarding P/LP disease variants results returned*		
*ID*	*Gender*	*Age*	*Children*	*Summary of health-related results and raw data returned*	*Nomenclature*	*Associated disease*	*Interpretation*
06	Female	40–44 years	0	0 P/LP disease; 3 P/LP carrier; e3/e3; T2D 1.45; CAD 0.77; AMD not displayed; raw data Y	—	—	—
							
**07**	**Female**	**55**–**59 years**	**0**	**3 LP disease**; 7 P/LP carrier; e3/e3; T2D 1.59; CAD 1.07; AMD not displayed; raw data Y	NM_000465.2(***BARD1***):c.1977A>G (p.R659=)	Hereditary breast & ovarian cancer	Likely pathogenic (LP) at time of study; re-classified as VUS after study completion
					NM_006393.2(***NEBL***):c.180G>C (p.K60N)	Dilated cardiomyopathy	Likely pathogenic (LP) at time of study; re-classified as VUS after study completion
					NM_003098.2(***SNTA1***):c.770C>G (p.A257G)	Long QT syndrome	Likely pathogenic (LP) at time of study; re-classified as VUS after study completion
							
08	Female	60–64 years	0	0 P/LP disease; 1 P/LP carrier; e3/e3; T2D 1.41; CAD 0.94; AMD not displayed; raw data Y	—	—	—
							
11	Male	60–64 years	1	0 P/LP disease; 3 P/LP carrier; e3/e4; T2D 0.14; CAD 1.08; AMD 0.82; raw data Y	—	—	—
							
**12**	**Male**	**50**–**54 years**	**0**	**1 LP disease**; 3 P/LP carrier; e3/e3; T2D 1.32; CAD 0.71; AMD 0.82; raw data Y	NM_000350.2(***ABCA4***):c.5908C>T (p.L1970F)	Macular degeneration	Likely pathogenic (LP) at time of study; re-classified as VUS after study completion
							
13	Male	50–54 years	2	0 P/LP disease; 3 P/LP carrier; e3/e4; T2D 0.97; CAD 1.26; AMD 1.04; raw data N	—	—	—
							
14	Female	60–64 years	0	0 P/LP disease; 2 P/LP carrier; e3/e4; T2D 0.61; CAD 0.73; AMD 1.00; raw data Y	—	—	—
							
**15**	**Male**	**25**–**29 years**	**0**	**1 LP disease**; 2 P/LP carrier; **e4/e4**; T2D 1.35; CAD 1.02; AMD 0.45; raw data Y	NM_198056.2(***SCN5A***):c.5770G>A (p.A1924T)	Brugada syndrome	Likely pathogenic (LP) at time of study; re-classified as VUS after study completion
							
16	Female	35–39 years	0	0 P/LP disease; 2 P/LP carrier; e3/e3; T2D 1.27; CAD 1.11; AMD 0.45; raw data Y	—	—	—
							
17	Male	50–54 years	2	0 P/LP disease; 1 P/LP carrier; e3/e4; T2D 1.21; CAD 1.01; AMD 1.24; raw data N	—	—	—
							
18	Male	60–64 years	0	0 P/LP disease; 0 P/LP carrier; e3/e3; T2D 1.19; CAD 1.17; AMD 1.21; raw data Y	—	—	—
							
**19**	**Female**	**45**–**49 years**	**2**	**1 P disease**; 2 P/LP carrier; e3/e3; T2D 1.47; CAD 0.81; AMD 1.19; raw data Y	NM_002016.1(***FLG***):c.2282_2285delCAGT (p.S761CfsX36)	Ichthyosis vulgaris, dry skin condition (semi-dominant)	Pathogenic (P)
							
**20**	**Male**	**25**–**29 years**	**0**	0 P/LP disease; 5 P/LP carrier; e3/e3; T2D 0.83; CAD 0.87; **AMD 2.83**; raw data Y	—	—	—
							
22	Female	50–54 years	2	0 P/LP disease; 2 P/LP carrier; e3/e3; T2D 1.34; CAD 0.71; AMD 0.75; raw data N	—	—	—
							
**23**	**Male**	**45**–**49 years**	**0**	**1 LP disease**; 3 P/LP carrier; e3/e3; T2D 1.20; CAD 0.94; AMD 0.39; raw data Y	NM 000041.2(***APOE***):c.460C>A (p.R154S)	Familial combined hyperlipoproteinemia type III	Likely pathogenic (LP)
							
24	Male	55–59 years	2	0 P/LP disease; 3 P/LP carrier; e3/e3; T2D 0.73; CAD 1.16; AMD 0.39; raw data Y	—	—	—
							
**26**	**Male**	**35**–**39 years**	**1**	0 P/LP disease; 0 P/LP carrier; e3/e3; T2D 0.82; CAD 1.14; **AMD 8.98**; raw data Y	—	—	—
							
27	Female	50–54 years	2	0 P/LP disease; 1 P/LP carrier; e3/e3; T2D 1.20; CAD 0.67; AMD 1.17; raw data Y	—	—	—
							
29	Female	30–34 years	0	0 P/LP disease; 2 P/LP carrier; e3/e3; T2D 0.73; CAD 0.77; AMD 0.44; raw data Y	—	—	—
							
30	Female	55–59 years	0	0 P/LP disease; 4 P/LP carrier; e3/e3; T2D 0.75; CAD 0.85; AMD 1.05; raw data Y	—	—	—
							
31	Male	25–29 years	0	0 P/LP disease; 2 P/LP carrier; e3/e4; T2D 0.65; CAD 1.14; AMD 0.65; raw data Y	—	—	—
							
**32**	**Male**	**35**–**39 years**	**1**	0 P/LP disease; 0 P/LP carrier; **e4/e4**; T2D 1.77; CAD 0.81; AMD 0.39; raw data Y	—	—	—
							
34	Male	65–69 years	2	0 P/LP disease; 2 P/LP carrier; e3/e4; T2D 1.23; CAD 0.92; AMD 0.39; raw data Y	—	—	—
							
35	Female	55–59 years	0	0 P/LP disease; 2 P/LP carrier; e3/e3; T2D 1.24; CAD 1.15; AMD 0.65; raw data Y	—	—	—
							
36	Male	45–49 years	2	0 P/LP disease; 2 P/LP carrier; e3/e3; T2D 1.38; CAD 0.91; AMD 0.46; raw data Y	—	—	—
							
37	Female	30–34 years	0	0 P/LP disease; 3 P/LP carrier; e3/e3; T2D 0.87; CAD 1.66; AMD 0.46; raw data Y	—	—	—
							
**38**	**Male**	**45**–**49 years**	**1**	**1 P disease**; 1 P/LP carrier; e3/e4; T2D 1.03; CAD 0.96; AMD 1.02; raw data Y	M_002016.1(***FLG***):c.2282_2285delCAGT (p.S761CfsX36)	Ichthyosis vulgaris, dry skin condition (semi-dominant)	Pathogenic (P)
							
39	Male	55–59 years	3	0 P/LP disease; 5 P/LP carrier; e3/e2; T2D 0.69; CAD 1.07; AMD 0.65; raw data Y	—	—	—
							
**40**	**Male**	**65**–**69 years**	**0**	**1 P disease**; 0 P/LP carrier; e3/e3; T2D 0.58; CAD 1.14; AMD 0.15; raw data Y	NM_198578.3(***LRRK2***):c.6055G>A (p.G2019S)	Parkinson's disease, late-onset (incomplete penetrance)	Pathogenic (P)

Abbreviations: AMD, age-related macular degeneration genetic risk score; CAD, coronary artery disease genetic risk score; N, no; T2D, type 2 diabetes genetic risk score; Y, yes.

Total *n*=29. Bold font highlights participants who received P/LP rare disease-associatd variants results, *APOE* e4/e4 results indicating increased risk of Alzheimer's disease, and/or AMD GRS-based relative risk scores greater than 2.0.

**Table 3:** Summary of results returned and participants' reactions by results category: qualitative outcomes

*Personal genomic results categories*	*Participants* *who received* *each result*		*Summary of participants' responses in the qualitative 6-month follow-up interviews*
	*N*	*%*	
*Rare disease variants*			
Pathogenic variant(s) Likely pathogenic variant(s) Pathogenic and likely pathogenic variant(s)No pathogenic/likely pathogenic variants	3 4 0 23	10.3 13.8 0.0 79.3	Two of the 7 participants who received ‘pathogenic' or ‘likely pathogenic' (P/LP) rare disease variant results were concerned about and had acted on their results. The first (#15, male, 25–29 years) had made an appointment and had a consultation, including tests and procedures, with a healthcare provider, as a direct consequence of his *SCN5A* Brugada-associated result; the second (#07, female, 55–59 years) had sought further information from family about her *NEBL* and S*NTA1* results, and this had then prompted her to also have a cardiac consultation with a healthcare provider. Both had concerns about the implications for health and insurance, and had sought further information online. The other 5 participants who received P/LP disease variant results had little emotional or behavioural reactions to these. Participants who did not have any rare disease variants identified in their results reports had little emotional or behavioural reaction to these results.

*Alzheimer's disease*			
*APOE* e4/e4 * APOE* e3/e4 * APOE* e3/e3 * APOE* e3/e2	2 7 19 1	6.9 24.1 65.5 3.4	Both participants who received *APOE* e4/e4 results were concerned. One (#15, male, 25–29 years) had also received the *SCN5A* Brugada-associated disease variant: he initially did not focus on the e4/e4 result as he was more concerned about the *SCN5A* variant; however, over time, he became less focused on this, and more concerned about the Alzheimer's risk. The other (#32, male, 35–39 years) was concerned, although he was happy about the experience in general, and very excited by the science. Neither had discussed their results with a doctor. Although there was little impact of the e3/e4 results on most participants, one was concerned (#11, male, 60–64 years): he had sought further information online, but this had not reduced his confusion and he remained concerned; he had not discussed the result with a doctor, in part due to insurance concerns; he had started to use a brain-training app. Some participants who received e3/e3 or e2/e3 results expressed relief; many did not react particularly to the result.

*Type 2 diabetes*			
Lifetime risk estimate: gender+age	18.9% to 45.2%		These results had little emotional or behavioural impact on most participants. A few participants made lifestyle changes in response to increased risk results.
24 variants GRS-based relative risk	0.58 to 1.77		
Lifetime risk estimate: gender+age+GRS	11.0% to 55.5%		

*Coronary artery disease*			
Lifetime risk estimate: gender+age	25.0% to 50.0%		These results had little emotional or behavioural impact on most participants. Very few participants made lifestyle changes in response to increased risk results.
29 variants GRS-based relative risk	0.67 to 1.66		
Lifetime risk estimate: gender+age+GRS	19.2% to 62.7%		

*Age-related macular degeneration*			
Lifetime risk estimate: gender+age 5 variants GRS-based relative risk Lifetime risk estimate: gender + age + GRS	8.0% to 25.0% 0.15 to 8.98 1.2% to 71.9%		Most participants received RRs <2.0 and most did not react emotionally or behaviourally to these results. Two participants received RRs > 2.0. One of these participants (#20, male, 25–29 years) had a relevant family history, and had shared their result with their doctor at a pre-scheduled appointment (RR 2.83); the other (#26, male, 35–39 years) planned to share the result with an ophthalmologist but had not done so yet (RR 8.98).

*Rare carrier variants*			
Pathogenic variant(s) Likely pathogenic variant(s) Pathogenic and likely pathogenic variant(s)None	16 2 7 4	55.2 6.9 24.1 13.8	There was little impact of P/LP disease carrier results on most participants who received these results. A few said that the information might be useful for their own, or for their children's, reproductive decision-making in the future. Some felt it was too much information and not relevant to them, and that they should perhaps have opted out of this section. Participants with no rare disease carrier variants identified had little emotional or behavioural reaction to these results.
			
*Pharmacogenomics*			
Clopidogrel (Plavix)			
Extensive metabolizer	11	37.9	Some participants felt that this information was *interesting*, and some felt that it might be useful to them in the future. A few would have liked information about more medications, including antidepressants.
Ultrarapid metabolizer	9	31.0	
Intermediate metabolizer	9	31.0	
Simvastatin (Zocor)			
Normal activity	19	65.5	
Intermediate activity	8	27.6	
Low activity	1	3.4	
Insufficient information	1	3.4	
Warfarin (Coumadin)			
***	***	***	

*Physical traits*			
Bitter tasting type Bitter taster Not bitter taster Insufficient information	17 7 5	58.6 24.1 17.2	Most participants did not react to these results. A few said they were interesting or fun.
Cilantro tasting type			
Less soapy taste	15	51.7	
More soapy taste	3	10.3	
Least soapy taste	3	10.3	
Insufficient information	8	27.6	
Earwax type			
Wet earwax	27	93.1	
Dry earwax	2	6.9	
Lactose intolerance			
Not lactose intolerant	12	41.4	
Possibly lactose intolerant	11	37.9	
Insufficient information	6	20.7	

*Ancestry (largest percentage)*			
Ashkenazi Jews Utahn Whites N European Spaniards	9 6 3 3	31.0 20.7 10.3 10.3	Many participants found these results interesting, and some found them fun. Many shared these results with their family and friends. A few said their results made them feel more connected to the world.
French	2	6.9	
Armenians	1	3.4	
African American	1	3.4	
Basque	1	3.4	
Bamoun	1	3.4	
Singapore Chinese	1	3.4	
Maya	1	3.4	

*Raw sequence data*			
Yes No	26 3	89.7 10.3	Several participants felt that their raw sequence data might be useful to them clinically in the future if they got sick.
