# Supplementary material for: Psychological and behavioural impact of returning personal results from whole-genome sequencing: the HealthSeq project
Source: Eur J Hum Genet. 2017 Jan 4;25(3):280–92. doi: 10.1038/ejhg.2016.178 (PMC5315514; doi:10.1038/ejhg.2016.178)
Supplement: Supplementary Figure 1 [file ejhg2016178x1.ppt]

## Slide 1
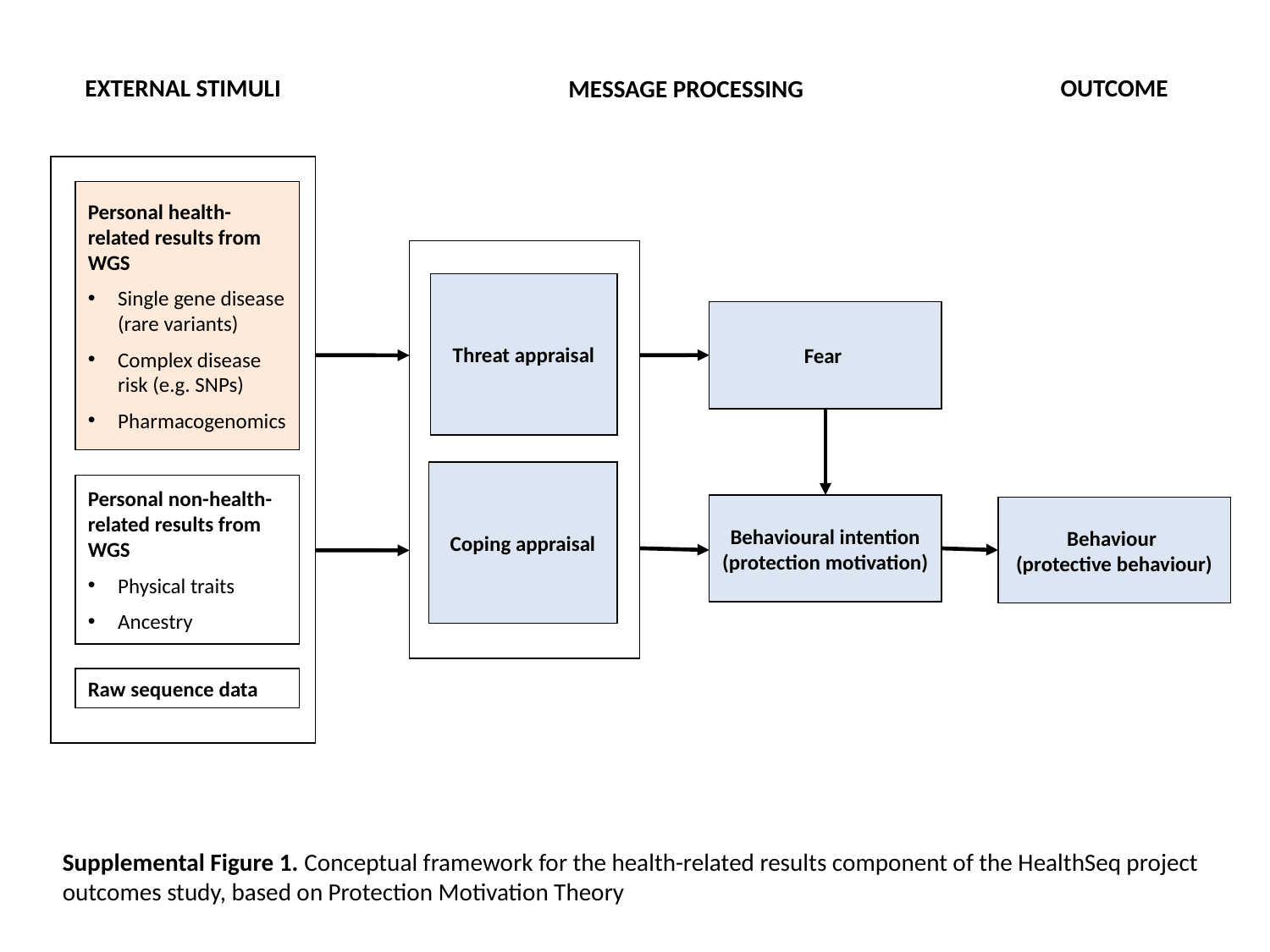

EXTERNAL STIMULI
OUTCOME
MESSAGE PROCESSING
Personal health-related results from WGS
Single gene disease (rare variants)
Complex disease risk (e.g. SNPs)
Pharmacogenomics
Threat appraisal
Fear
Coping appraisal
Personal non-health-related results from WGS
Physical traits
Ancestry
Behavioural intention (protection motivation)
Behaviour
(protective behaviour)
Raw sequence data
Supplemental Figure 1. Conceptual framework for the health-related results component of the HealthSeq project outcomes study, based on Protection Motivation Theory
