# Supplementary material for: Psychological and behavioural impact of returning personal results from whole-genome sequencing: the HealthSeq project
Source: Eur J Hum Genet. 2017 Jan 4;25(3):280–92. doi: 10.1038/ejhg.2016.178 (PMC5315514; doi:10.1038/ejhg.2016.178)
Supplement: Supplementary Tables 1 to 5 [file ejhg2016178x2.docx]

**Supplemental Table 1.** Constructs included in the HealthSeq project and whether they were assessed using qualitative or quantitative methodological approaches

|  | **Measurement type** | |
| --- | --- | --- |
| **Construct** | **Qualitative** | **Quantitative** |
| **PMT constructs** |  |  |
| Threat appraisal (perceived susceptibility) | ✓ | 🗶 |
| Coping appraisal (perceived response-efficacy) | ✓ | 🗶 |
| Fear^a,e^ | ✓ | ✓ |
| Behavioural intention^e^ | ✓ | 🗶 |
| Behaviour^b,e^ | ✓ | ✓ |
| **Non-PMT constructs** |  |  |
| Positive emotional responses to results^c,e^ | ✓ | ✓ |
| Uncertainty about results^d^ | ✓ | ✓ |
| Perceived value of results^e^ | ✓ | 🗶 |
| Depression^e^ | 🗶 | ✓ |
| Anxiety^e^ | 🗶 | ✓ |
| Quality of life^e^ | 🗶 | ✓ |

^a^ Fear was operationalized in the quantitative questionnaire as test-related distress, assessed using the distress subscale of the MICRA

^b^ Actual behaviours assessed in the quantitative questionnaire were limited to lifestyle behaviours only

^c^ Uncertainty about results was assessed in the quantitative questionnaire using the uncertainty subscale of the MICRA

^d^ Positive emotional responses to results was assessed in the quantitative questionnaire using the positive experiences subscale of the MICRA

^e^ Included in the analyses for the present paper

**Supplemental Table 2.** Positive psychological reactions to personal results from whole-genome sequencing at 6-month follow-up: themes and example quotes

| **Themes** | **Sub-themes** | **Example quotes** |
| --- | --- | --- |
|  |  |  |
| **1. Results made them feel happy or relieved** | **1.1. Happy about their experience in general** | *“Oh, I’m super happy that I did it. Yeah. No, I wanted to do it. I do feel really good about having done it.”* (#08, female, 60-64yrs) |
|  | **1.2. Felt that they learned something new** | *“Generally I was really glad I took part in it ‘cause I feel like I learned a lot, which I’m always trying to do.”* (#08, female, 60-64yrs) |
|  | **1.3. Excited by the science** | *“I’m geeked. I’m still geeked. I was geeked at the beginning. I’m geeked now. I love it. I think it’s one of the best things I ever did.”* (#32, male, 35-39yrs) |
|  | **1.4. Relieved about their results for their own health** | *“I feel good because my results were pretty benign. I mean I didn’t have any results that made me feel nervous, would make me cautious. I feel good.”* (#30, female, 55-59yrs)  *“Well, very relieved. Overall, just very relieved. …Just a huge sigh of relief.”* (#08, female, 60-64rs) |
|  | **1.5. Relieved about their results for their children’s health** | *“The biggest thing that I was afraid of was that I was gonna be a carrier of something that was gonna go to my children, so luckily that wasn’t the case. I was definitely happy about that, but otherwise I felt okay.”* (#26, male, 30-35yrs) |
|  | **1.6. More optimistic about the future** | *“I’m glad I did it. … I was going through a phase a few years ago where I felt old and so…, I dunno, I was turning 50, or whatever. I really felt like everything was downhill from here [laughter]. For some reason, which may not make much sense, this thing made me feel more optimistic. Yeah, because I felt like, oh, there are not really any bad markers. The stuff that came up is stuff I expected. Okay. Maybe I should just calm down a little bit [laughter], and not feel so ancient.”* (#35, female, 55-59yrs) |
|  | **1.7. Felt they now know more about themselves** | *“Well, I know more about myself than before the results. That’s pretty much what I wanted out of it – to know more about me, to know more about myself.”* (#29, female, 30-34yrs) |
| **2. Experience or results were interesting** | **2.1. Found the experience overall interesting** | *“Just interesting to know that we can do that now, and, you know, I think it’s kinda cool because not that many people have done it, but I’m also a scientist. [Laughter] To me it was just interesting.”* (#37, female, 30-34yrs) |
|  | **2.2. Found the pharmacogenomics results interesting** | *“In terms of other things—I mean some of them were—there was kind of an interesting thing about how you could predict your—I believe it was a blood clotting thing based upon the genotype. That was actually quite interesting. I mean, I hope I don’t—need to go on warfarin or whatever at some point. It looked like it was actually a place to start your dose level, based upon the genotype.”* (#34, male, 65-69yrs) |
|  | **2.3. Found the ancestry results interesting** | *“That [my ethnic heritage] was very interesting to me because there were certain things in there, which certainly none of my parents had any idea about. That was fascinating.”* (#19, female, 45-49yrs) |
| **3. Glad to have made a contribution to research** |  | *“Oh, I’m feeling very good about it. I’m glad to have been able to participate in this and be helpful in some way.”* (#18, male, 60-64yrs) |
| **4. Results or data might be useful in the future** | **4.1. Results may be useful to them clinically in the future** | *“I know that if I get sick in certain ways that there’s information on the hard disk that will help my doctors see interactions and efficacies. I know that, and I’m very grateful for that.”* (#24, male, 55-59yrs) |
|  | **4.2. Results may be empowering to them in the future** | *“It was stressful in the short-term. … I think going forward I’ll be concerned about the risk of sudden cardiac death, but I think hopefully this information will be empowering.”* (#15, male, 25-29yrs) |
|  | **4.3. Results may be useful to them or their children for reproductive decision-making in the future** | *“The fact that we do know about the two diseases that I carry, so that in 15 years or whatever it is or 20 years when either of my kids decides to have a child that that’s something we can already be aware about. … It was more comforting than anything. … It was more comforting and interesting than anything else.”* (#19, female, 45-49yrs) |
| **5. Results made them feel more connected to the world** |  | *“Well, certainly the ethnic genetic distribution chart was… I found fascinating and absorbed the meaning of it over the last few months. It changed my view of myself, in a way. I had certainly considered myself primarily European. In fact, the genetic markers seem to indicate that there are a variety of other ethnic backgrounds in my genes. I certainly don’t know how they got there, but it increases my appreciation of how interconnected we all are. [Laughter] I mean, it was interesting to find out that I’m a couple percent Iraqi Jewish, which means that I… it slightly changes my view of what’s going on in Iraq these days because suddenly I feel like I have an ethnic or historical connection to a country that, before, was somewhat… I was somewhat disengaged from the actual horrors that are going on there. I mean, it helps one feel more connected to places and people that seemed not to have a connection to me before… It was surprising, and fascinating, and just enriching of my sense of myself and my place in the world.”* (#18, male, 60-64yrs) |
| **6. Results were fun** |  | *“Yeah, I told my, I was telling my parents about it, some of my friends. Mostly people were interested about the ancestry part. That’s the whole reason – fun thing [is] that you get unexpected results.”* (#26, male, 35-39yrs) |

**Supplemental Table 3.** Negative psychological reactions to personal results from whole-genome sequencing at 6-month follow-up: themes and example quotes

| **Themes** | **Sub-themes** | **Example quotes** |
| --- | --- | --- |
| **1. Concern** | **1.1. Concerned about the implications for their health** | *“It was pretty concerning to me because it is a sudden cardiac death mutation, so it’s one of those things that [pause]—it really can strike at any time, and it’s—so I was scared for that reason.”* (#15, male, 25-29yrs)  *“I was like really concerned about the Alzheimer’s stuff… I’m still kind of, I don’t know, worried about it.”* (#11, male, 60-64yrs)  *“All I know that it [type 2 diabetes risk] is above the normal population range. It’s something that I’m taking very seriously, because I know that that could be an issue in the future. I was very concerned.”* (#06, female, 40-44yrs). |
|  | **1.2. Concerned about the implications for insurance** | *“My major concern was going through insurance and then having the information which was done in a research setting but confirmed in a clinical laboratory, but having that put into my records, like my clinical records, was very concerning…”.* (#15, male, 25-29yrs)  *“My physician here, she’s all into the chart… she’s very conservative like that with the medical records so I’m not telling her because I don’t want her writing down, “He’s at risk for Alzheimer’s,” I don’t want that…. I don’t trust insurance companies, that’s for sure.”* (#11, male, 60-64yrs) |
| **2. Disappointment** | **2.1. Had hoped to get information about a specific disease or trait** | *“I wish it would have flagged out about my color-blindness… I would be interested to see what my genes say my risk of color-blindness should be versus what it is.”* (#17, male, 50-54yrs) |
|  | **2.2. The results didn’t meet their expectations** | *“I left the whole process feeling disappointed a little bit in terms of what I actually learned…. I think my feelings of disappointment were related to where the technology is.”* (#13, male, 50-54yrs) |
| **3. Indifference** |  | Interviewer: *“How do you feel about receiving this information?”* Interviewee: *“Indifferent… Maybe if I had some evidence of the problems, like heart disease or Alzheimer’s, or something like I needed some of the drugs that were mentioned on the pharmacogenetics thing… I’m in good health, it doesn’t really [inaudible] at this point.”* (#31, male, 25-29yrs) |
| **4. Confusion** |  | *““If I forget things I already say to myself, “Gee, I have that E34 maybe—” I don’t know, so it’s kinda—I don't know, it’s not a big deal. Though I mostly think like I'm probably not going to get Alzheimer's disease, yeah, but the information, having received it, is sort of like, it’s a little concerning in a way…. Then there’s so much stuff on the internet, I have no idea what’s right and what’s wrong.”* (#11, male, 60-64yrs) |

| **5. Desire for more results** | **5.1. Medications in general** | *“Yeah, how you response to drugs. Yeah, that was interesting, I remember. You were only looking at three kinds of drugs… I mean, I just remember thinking it’d be more interesting if you had more drugs. Mine were all like average response or whatever.”* (#31, male, 25-29yrs) |
| --- | --- | --- |
|  | **5.2. Antidepressants specifically** | *“The three or four things that they picked, or the medication, the statins might not be great for me, that was interesting…. The only [thing] I would say was I thought there was going to be a lot more information than there was. [The genetic counselor] explained, ‘Well, we just don’t have stuff on every medication.’ Oh, I know what I wanted, I wanted what was the optimal antidepressant for me genetically. She said, ‘Well, we just don’t have that information.’ That would have been great to know.”* (#11, male, 60-64yrs) |
| **6. Results not relevant to them** |  | *“I think it was almost like too much data. When I was getting the results, you know, during the interview, or during the counseling session, it was almost like too much. I think in retrospect I think I would have skipped the whole carrier gene section. Because, you know, I’m past child-bearing age. I don't have children. It was almost too much to take in in one session. I don't know if that was even worth the time spent.”* (#07, female, 55-59yrs) |

**Supplemental Table 4.** Behavioral reactions to personal results from whole-genome sequencing at 6-month follow-up: qualitative themes and example quotes

| **Themes** | **Sub-themes** | **Example quotes** |
| --- | --- | --- |
| **1. Shared results with family and friends** |  | *“I shared it with some of my friends, just close friends. I shared it with my boyfriend, my brother, my mom. Just very close people to me.”* (#37, female, 30-34yrs) |
| **2. Sought further information** | **2.1. Sought further information from family** | *“Then the other piece is just the two mutations related to heart disease. … That was enough for me to try to find out more about the cardiac history in my family. That was a little surprising. …”* (#07, female, 55-59yrs) |
|  | **2.2. Sought further information online** | *“I did look up the three mutations, the BARD1, and then the two cardiac ones. The one for Long QT syndrome, and then the other one for dilated cardiomyopathy, just to understand a little more about what it’s about. Based on the descriptions it didn’t sound like just from a cardiac standpoint that I had it.”* Interviewer: *“How did you look into those? What kind of resources did you use?* Interviewee: *“Just Wikipedia, I think.”* (#07, female, 55-59yrs)  *“I did look into the ancestry more. There were some ethnicity there that I wasn’t really familiar with, so I did look them up.”* (#29, female, 30-34yrs) |
| **3. Shared (or not shared) results with a healthcare provider** | **3.1. Mentioned results to their healthcare provider at a pre-scheduled appointment** | *“I brought it up in the context of my primary care. She said it was good that I’d got it done, and is being very aggressive about making sure that she follows up in relation to this.”* (#06, female, 40-44yrs)  *“I did tell her [about the risk of AMD result]…. When she was just going through and doing just the basic family history. She asked. I went through the kind of standard stuff that I had always told her, and then I said also in addition to this I had my genome sequenced recently here. This is information that came from it that I think is relevant to include.”* (#20, male, 25-29yrs) |
|  |  | “*Yeah, I did mention some of the results to my physician when I happened to be in…. I think I was just in for bronchitis, but we were just chatting about that.”* (#39, male, 60-64yrs) |
|  |  | *“I mentioned it… I just mentioned that I had it done but we didn’t discuss any of that, all the details…. Last October, I had an annual checkup. He just asked me general questions and I said, I mentioned it to him.”* (#34, male, 65-69yrs) |
|  | **3.2. Had their results put in their medical record** | *“I have shared the entire report with my primary care physician…. He has a record of it…. I gave him the hard copy, but he is very electronic record oriented. I think he will—he was gonna have his people scan it and attach it to my medical record.”* (#18, male, 60-64yrs) |

|  | **3.3. Made appointment and had consultation, including follow-up tests/procedures, with a healthcare provider** | *“I’ll find out Friday about the cardiac test… While I had the initial meeting with the cardiologist he suggested also losing weight. It’s kind of hand in hand.”* (#07, female, 55-59yrs)  *“So I was able to meet with Dr [name], and he did the EKG, which didn’t find anything pathological, and then we talked about it [pause] without putting all the information in my—all the detailed clinical information in my chart,* *able to reassure me and provide some advice, but mostly saying that I should not worry about it all too much… After talking to him, I was reassured of it, and that has slowly let it drop…”* (#15, male, 25-29yrs) |
| --- | --- | --- |
|  | **3.4. Intends to make appoint with a healthcare provider but has not done so yet** | *“I think it was like in the 70-75, something like that, so that was a high risk. I was planning to go to an ophthalmologist. I just haven’t had a chance to do that.”* (#26, male, 35-39yrs) |
|  | **3.5. Not shared -- Didn’t think their physician would understand** | *Well, I don’t think that my physicians would be equipped to do anything with that genetic information.”* (#07, female, 55-59yrs) |
|  | **3.6. Not shared -- Thought physicians were too busy** | *“I don’t think most physicians are interested anyway… It’s just physicians are overworked. They don’t have that much time… It consumes so much time.”* (#31, male, 25-29yrs) |
|  | **3.7. Not shared -- Didn’t think there was anything to discuss** | *“I don’t think there was really anything there that was—needed discussing”* (#19, female, 25-29yrs) |
|  | **3.8. Not shared -- Didn’t know what type of physician to consult** | *“I don’t know who treats Alzheimer’s disease, a neurologist? Or is it psychiatry, I don’t know.”* (#11, male, 60-64yrs) |
|  | **3.9. Not shared -- Didn’t think it would change their care** | *“I didn’t share with my doctor, and I kinda wanted to, but I kinda went back and forth about it; just, I don’t know, just didn’t… I wasn’t sure what it would really bring to anything that isn’t already there in the first place. …she’s already worried about heart disease and diabetes and all those things for me in the first place.”* (#32, male, 35-39yrs) |
| **4. Made lifestyle changes** | **4.1. Started exercising** | *“It was enough for me to consider losing a few pounds. I have started exercising this month. Joining a gym, it’s been a while. I’m really trying to get with the program”* (#07, female, 55-59yrs) |
|  | **4.2. Made dietary change** | *“Knowing that there was, there were other coronary or heart related risks, this certainly supported my efforts over the last six months to lose weight and eat more healthy, eat healthier, as has the knowledge that I have a more, better than average risk of developing, I guess, adult-onset diabetes.”* (#18, male, 60-64yrs) |
|  | **4.3. Started to use a brain-training app** | *“Yeah, I joined Lumosity, this brain game thing. Look it up; Lumosity. It’s supposed to be based on research, medical research, literally helps retention span, the brain activity, there’s all these challenging puzzles, games. Like reaction games and shapes.”* (#11, male, 60-64yrs) |

**Supplemental Table 5.** Lifestyle health behaviours and quality of life at baseline and 6-month follow-up: quantitative outcomes

| **Variable** | **Baseline (T1), N (%)** | **6-month follow-up (T4), N (%)** | **Sig.** |
| --- | --- | --- | --- |
| **Lifestyle health behaviours:** |  |  |  |
| Diet: Do you eat five or more servings of fruit or vegetables a day? *Yes* | 17 (58.6%) | 17 (58.6%) | Z=0.00, p=0.99 |
| Vitamins: Are you taking any vitamin supplements? *Yes* | 9 (31.0%) | 9 (31.0%) | Z=0.00, p=0.99 |
| Alcohol: How many servings of alcohol do you have on a typical day? *One or more* | 11 (37.9%) | 14 (48.3%) | Z=1.67, p=0.096 |
| Physical activity: Do you walk (or do moderate activity) for at least 30 minutes on most days, or at least 3 hours per week? *Yes* | 26 (89.7%) | 23 (79.3%) | Z=1.32, p=0.18 |
| Physical exercise: During the past month, other than your regular job, did you participate in any physical activities or exercises such as running, golf, gardening, or walking for exercise? *Yes* | 26 (89.7%) | 26 (89.7%) | Z=0.00, p=0.99 |
| Smoking: Do you smoke cigarettes – *every day or some days* | 4 (13.7%) | 6 (26.7%) | Z=1.22, 0.22 |
| **Quality of life:** |  |  |  |
| Self-rated health? Excellent or very good | 24 (81.7%) | 21 (60.0%) | Z=1.90, p=0.058 |
